# On the Premonitory Diarrhœa of Cholera

**Published:** 1855-12

**Authors:** George Todd

**Affiliations:** Surgeon, West Auckland.


### ON THE PREMONITORY DIARRHŒA OF CHOLERA.

By George Todd, Surgeon, West Auckland.

"He who, whether by original observations, or by legitimate deduction drawn from the observations of others, shows the identity of, or points out an analogy between disorders hitherto commonly thought to be distinct and dissimilar complaints, by so far as he accomplishes this, does he assist in simplifying medicine, and, consequently, contributes towards its advancement, theoretically as well as practically."

It has been generally observed as a fact, ever since epidemic cholera became known to the medical profession, that the greater proportion of cases are preceded by a distinct premonitory stage, varying, however, in duration and intensity, from slight disturbance in the functions of the alimentary canal, and gradually progressing to the production of the characteristic choleraic rice-water evacuations; and in duration, from several days to a few hours, or even to one hour's duration, before the full development of the graver symptoms of this disease. Cases

---

no doubt do occur in which this premonitory stage is of such short duration as not to attract notice; and such cases have frequently been brought before the medical profession as proofs of cholera without any premonitory diarrhœa; such cases will, however, I am of opinion, be found on inquiry to have been preceded by a well-marked premonition.

From July 1853 to the 11th of February 1854, 878 deaths were registered of cholera. Out of this number twenty-one cases were reported to the registrar-general as cases of cholera without any premonitory diarrhœa. Dr. Macloughlin made it his duty—and a very important one to the profession—to see the relatives and attendants on the last moments of these twenty-one cases, in order to ascertain the correctness of the returns made. The result of this very important investigation was, that fifteen of them had a diarrhœa for some hours, or for some days, previous to the attack of spasms, vomiting, purging, etc. The remaining six cases were either not cases of cholera, or cases where nothing was known of their previous history, proving, as Dr. Macloughlin justly remarks, the superficial manner in which too often medical gentlemen make their investigations at the bed-side, with regard to that most important and essential point, the state of their patients previous to their being summoned to their assistance.

If any doubt could exist during the epidemic of 1832 as to the general and, I may say, universal prevalence of a premonitory stage of diarrhœa, the experience of the later epidemics must have settled this question. There is ample evidence to prove that over Europe, and in every town and village of this country where cholera has appeared, it was invariably preceded by a premonitory diarrhœa. In Russia, wherever cholera was present, diarrhœa existed; and in Berlin, it is said, that almost every person was affected in the same manner. In Hamburg also, great numbers laboured under a premonitory diarrhœa. In London, during the epidemic of 1848 and 1849, there was an enormous amount of this premonitory affection of the bowels. In other towns, Bristol, Hull, Manchester, and Liverpool, the same general prevalence of diarrhœa was observed. In Scotland likewise, it was said, that nearly the whole population were thus affected.

During the epidemic of 1831-2, this premonitory diarrhœa did not escape the notice of the medical men of this country; and a large amount of concurrent testimony might be cited as to the universal prevalence of a premonitory stage, and the very important and absolute necessity of directing treatment specially against it.

During this epidemic of 1831-2, I had charge of a cholera district near Newcastle-upon-Tyne, comprising Long Brenton, Killingworth, West Moor (a very populous colliery village), and their neighbourhoods; and I have notes of upwards of one hundred and fifty cases of cholera which I attended. About the decline of the epidemic, I was attacked with the disease myself, and narrowly escaped with my life. In all these cases, without a single exception, there was more or less of premonitory diarrhœa, in some of several days duration, in others, but of a few hours, and in two cases, which might have been reported as without any such premonitory stage, of very short duration. In one, where the disease commenced with fæculent purging, the characteristic rice-water stools made their appearance in two hours, and the patient died in twelve hours from the commencement of the attack, suffering frightful spasms up to the time of her death. I am enabled to speak from facts in reference to this case, as I was called to her on the first appearance of the premonitory symptoms, and I did not leave her till she died. I may add, that I examined the evacuations at the commencement of the disease; they were very copious, distinctly fæcal, and of a bilious appearance, the succeeding stools becoming gradually of a more and more serous and rice-water character as the disease progressed. In another case, that of a woman in the eighth month of pregnancy, the premonitory diarrhœa was not of more than three or four hours duration, and the patient sank from exhaustion, without any spasms, an hour or two after having been delivered by the forceps, there being no uterine action, or at least of a very feeble nature, and about six hours from the commencement of the premonitory and fæcal diarrhœa; for, in this case, as in the preceding, the serous and rice-water purging quickly followed the few fæcal stools at the commencement. My own case illustrates and confirms these facts. At the time I was attacked, I had not been one whole night in bed for a month, when I was called at midnight to visit two cases of cholera in one family. On my entering the house, the patients were both on the close-stool, or rather seated on two tubs, and the stench was overwhelming, and made an unusual impression upon me. After leaving the house, and on my way home on horseback, I felt a sensation of coldness, particularly over the abdomen, and a feeling as if the *whole abdominal viscera were falling down.* I was soon obliged to dismount, and had a most copious fæcal evacuation, leaving a sensation as if the intestines were completely emptied of their whole contents, but without any acute or griping pain; before I reached home, about a mile from the patients' house, I had five copious liquid stools; the diarrhœa continued for twenty-four hours, becoming more and more of a serous nature, but without, however, assuming the rice-water appearance. I was confined to bed for a fortnight, and reduced to the very last stage of debility. I believe that but for the very prompt and energetic treatment which I received during the premonitory stage of the disease, it would have progressed to its graver stage of collapse, and that my chance of recovery would have been doubtful indeed.

Thus there was a premonitory diarrhœa in all those cases; and, in the great majority, of from several days to several hours duration. The dejections were at first copious, fæculent, and of dark colour, becoming gradually paler, and afterwards colourless, like rice-water.

In June 1850, I was called to a fatal case of sporadic cholera, which still further illustrates and confirms the facts I have already stated as to the invariableness of a premonitory diarrhœa as the first stage of cholera. The case was that of a man, aged 50, previously in good health, who resided in the neighbouring village of Evenwood, and lived alone. He was attacked with diarrhœa early on the morning of the 10th of June, and a neighbour observed that he went to the privy very often during the day. I was requested to see him about six o'clock the same evening, and was told by a neighbour that she found him lying on the floor deluged in rice-water excreta. This I was able to verify, as the floor was covered with evacuations of this nature when I arrived. The patient was far advanced in the stage of collapse, and died about six hours afterwards. Thus this poor unfortunate patient had had a premonitory diarrhœa for several hours; which, during the time he was able to obey the calls of nature, had, in all probability, been of a fæculent character; as the strength failed, and rice-water purging succeeded, he became unable to reach the privy, and probably in making the attempt sank on the floor and was found in the state I have described. I examined the body the following day. The external appearances were characteristic of the disease: a livid surface; the skin of the hands and feet corrugated; the nails blue, and the fingers rigidly contracted. The lungs were collapsed, and deficient in crepitation to a very remarkable degree; the large vessels were filled with dark-coloured blood; the right side of the heart and the pulmonary arteries were filled with dark-coloured semi-coagulated blood; the left side and aorta contained only a small quantity of dark blood; there was some accumulation of blood in the larger branches of the vena portæ and hepatic vein; the gall-bladder contained much thick and viscid bile. The stomach contained about six ounces of a dark brown-coloured fluid. The small intestines contained a large quantity of white curdy fluid, of a thinner and a thicker portion; the thicker portion was curdy or clotted, and lay in masses here and there; the mucous membrane was soft and pulpy; the large intestines were empty; the mucous membrane presented several slight vascular patches. The head was not examined.

For the last three months, diarrhœa has prevailed here and in the neighbourhood to an extent which I have never before witnessed, and the few cases of confirmed cholera which I have seen during that time have invariably been preceded by some hours, a day, or some days of premonitory diarrhœa; indeed, the severity of the case has always been in proportion to the attention or neglect which had been paid to this premonitory purging; and I am quite confident that but for my early and prompt treatment of this important stage of the disease, I should have had many more cases of confirmed cholera. For instance, I have had one case, to which I was called after the patient had been suffering from violent premonitory diarrhœa for nearly twelve hours; and, on my visiting this patient, the rice-water evacuations were just commencing. I believe that if I had been two hours later in seeing this patient that all the different plans of treatment would have been unavailing; for I am confident that if the disease is allowed to progress beyond this most important—and in general curable—stage, all our efforts too often prove of no avail. How very important—nay, absolutely necessary—it is, therefore, to attack this malady in its first, and only curable, stage of what is called premonitory diarrhœa. Dr. Barry, no mean authority in such a case, said emphatically, in regard to the premonitory stage, "*Stop it, and you will save your patient.*" Dr. Sutherland, equally impressed with the importance of this premonitory stage, says that, in the earliest stages there is no disease more easily manageable, and in which so great an amount of human life and suffering can be saved; but in its later stages there is hardly a disease more completely beyond human control, and of which so large a proportion of cases must inevitably perish. Dr. James Adams says that a ratio was found to exist between the earliness of medical aid, and the result of the treatment. In cases which were brought under treatment within six hours of the time of attack, the per centage of deaths was only 21; between twelve and twenty-four hours, 45 per cent. died; when a delay of more than twenty-four hours took place before application was made for medical aid, the deaths rose to above 62 per cent. The conclusion must therefore be self-evident that the whole force of the medical treatment should be directed against this early stage of the disease.

This premonitory diarhœa is, I think, the first stage of the disease, and, consequently, no case of cholera can possibly exist without it. Dr. Sutherland, in his report to the Board of Health, says that strong evidence has been afforded of the unity of cholera throughout all its stages. So thoroughly, he says, has this fact been impressed on the minds of many eminent practitioners, that he has occasionally experienced considerable difficulty in obtaining statistical data, in consequence of its being found "impossible to draw any line between the most severe cases of cholera and the ordinary diarrhœa prevailing, warranted by any pathological distinction." If this premonitory diarrhæa be not the primary stage of the disease, the question may fairly be asked what is it? I have no hesitation in saying that this premonitory diarrhœa is as much a part of an epidemic of cholera as any one of the primary symptoms of continued fever is a part of the disease. It is quite true that these cases of premonitory diarrhœa, if promptly treated, may not be attended with equal peril to life; but there is abundant evidence to prove that in cholera, as well as that of continued fever, to which it bears a very close analogy, if not identity, the ratio of danger is determined by the promptness of the treatment during this primary stage, rather than by considering it with reference to any apparent difference in the causes. Whatever variety of opinion there may be on this most important point, it is practically impossible, and highly dangerous to the patient, to make any distinction. I wish to press on the minds of the profession, and more particularly so on the minds of those who may be engaged in treating this disease, that there ought to be no mistake on this point, as it is a matter of life and death.

The great majority of practitioners who have seen and treated cholera in all countries and under all varieties of conditions in which this disease has been epidemic, uniformly agree in reference to the premonitory diarrhœal stage. Some medical men who have treated this disease in India, state that the disease is extremely sudden—that frequently an individual is carried off in a few hours without any premonitory purging. In the Bengal report of 1820, it is stated that "the attack was generally ushered in by a feeling of fulness, pain in the stomach, and swelling of the abdomen, *with a desire to go to stool.* Then came almost immediately vomiting and purging of a pale thin fluid, &c." In some *rare* instances, it is said that the virulence of the disease was so powerful as to prove *immediately* destructive of life, as if the circulation was at once arrested. The patient fell down as if by lightning, and instantly expired. Those cases must have been of a similar nature to the one related in Dr. Macloughlin's pamphlet on cholera, where at page 17 it is said that a trumpeter of the 51st Regiment, in India, blew his trumpet for the officers' dinner so strong and so correctly as to be the subject of remark, and yet only a few minutes after he was seized with cholera and died before the officers' dinner was over! No inquiry, it appears, had been made as to this man having had any diarrhœa previous to the attack of spasms, &c. Many persons will actually believe themselves to be in perfect health up to the very moment at which they are seized with spasms, vomiting, and rice-water purging; and yet, as Dr. Macloughlin justly observes, these persons had a diarrhœa on them for a longer or shorter period, which they did not attend to, because it was painless. Various quotations could, however, be adduced from the writings of Indian practitioners, such as Dr. James Bird and Dr. Parkes, to prove that diarrhœa, in a vast majority of cases, precedes cholera in India.

In a very interesting discussion which took place at the Academy of Medicine at Paris, M. Guérin said that "cholera was almost always, if not always, preceded by a premonitory diarrhœa, and that it was extremely easy to arrest the disease at this period." Dr. Müller, in his account of the epidemic of 1848 at St. Petersburgh, says that premonitory signs, usually continuing for several days, and invariably connected with disturbance of the digestive organs, scarcely ever fail." It has been noticed by the Russian physicians that the precursory symptoms were invariably a painful or painless diarrhœa, which have all the ordinary fæcal characters, whilst the dejections in the subsequent stage resemble a decoction of rice. Dr. Neville says, that in the general hospital at Hamburg there was premonitory diarrhœa in every case, which was rapidly followed by the actual attack, death ensuing in a few hours. At Berlin, Dr. Romberg states that diarrhœa precedes the actual attack of cholera, and that he has not seen such sudden seizure as those described in India, without premonitory signs. During the epidemic cholera at Malta in 1837, Dr. G. Stilon says that the second stage, collapse, of the disease never occurred unless the diarrhœa had previously manifested itself for some time. In some cases, he says, "the individuals had suffered from this premonitory diarrhœa for twenty days, whilst in other cases it had lasted only a few hours previous to the second stage, and that the danger was in direct ratio of the greater or less evacuation of the *liquid fæces.*" And he further says, that "as diarrhœa was in Malta the *primary* and most remarkable symptom of cholera, the second stage was always preceded for a greater or less length of time by diarrhœa, some patients having suffered it for fifteen or twenty days before."

The experience furnished by the epidemic cholera, 1831-2, as well as of all the succeeding epidemics which have prevailed in this country, has amply confirmed the existence of this premonitory stage, and the comparative ease with which it may be treated; so that I think that it may now be considered an established fact, and that the cases where the diarrhœa is said to be absent, are cases where the disease, if any, has been superficially observed.

It is worthy of remark that now, when diarrhœa and cholera cases are subsiding in this neighbourhood, they are succeeded by what may prove to be a very extensive and malignant epidemic of continued fever; already fever has been fatal to four members of one family. It appears to be of the gastro-enteric type; but cases have occurred where the predominating symptoms have been located in the brain. Measles are also very prevalent in this locality. I am inclined to believe that there is a very great analogy, if not identity, in the cause producing cholera and fever.

It is certainly the opinion of a large number of medical observers who have seen the disease in India and other countries, as well as in England, that cholera bears a very close resemblance to fever of a remittent or continued type; it may therefore be asked whether its character is so much more closely related to that of fever as to render it advisable, consistently with accuracy of arrangement and precision of nomenclature, to place it among febrile disorders, and thus give it a designation more corresponding than its present one with its new nosological position? "If," as Dr. Brown justly says, "we embrace, as we ought to do, all the parts of cholera as we witness them in Europe,—if we consider that the choleraic symptoms, if not fatal, prove but the commencement of a series of changes to which anyone who witnessed them alone would give the appellation of fever, and which men of great experience in the disease have declared they could not distinguish from typhus,—if we observe, too, that long before this epidemic, 1832, excited attention, symptoms strikingly resembling those of cholera had been observed to form the initiatory stage of certain malignant fevers,—we are disposed to admit that it is in reality a fever, and that to designate it merely cholera"—a term signifying nothing—"is to take a part for the whole, and to give an erroneous idea of the disease." And he further says, that "he has not met with a single case of cholera in which fever did not intervene between the choleraic cold stage and restoration to health; and the experience of others most observing and familiar with the disease in this country, has coincided with his own on this point." I quite coincide with Dr. Brown's opinion on this point, and believe that the series or congeries of symptoms to which we give the name of cholera, is in truth nothing more than the aggravated effects of the same febrific poison upon a recipient incapable of coping with or reacting against it, which, when evolved from the earth or the atmosphere in a more dilute or less active state, or impinging on an individual possessing greater vital elasticity and reactive power, would produce an attack of continued fever, which is generally prevalent at, or subsequent to, the time of cholera.

Whatever may be the essential nature of cholera, it evidently belongs to the great epidemic class, and its habitat and activity bear a very close relation to continued fever, the causes which influence the one affecting the other. It appears, from Mr. Grainger's report on cholera to the Board of Health, that the epidemic of 1848-9 was preceded by a steadily increasing prevalence of fever in the Metropolis during the three years preceding cholera. From the registrar general's last quarterly report it appears that diarrhœa, fever, scarlatina, and measles, have prevailed very generally: at Heavitree, Devonshire, scarlatina, fever, and small-pox, have been prevalent and fatal. At Plymouth, it is stated, diarrhœa has been prevalent in all parts of the district throughout the summer. At Taunton, diarrhœa has been, and is now, very prevalent; scarlatina general, and frequently assuming a typhoid type. Several cases of low typhus have occurred. At Frampton, Gloucestershire, scarlatina and cholera have been prevalent. One of the cholera cases, it is said, was preceded by an attack of measles. At Darlaston, Staffordshire, measles, scarlatina, typhus, hooping-cough, and diarrhœa, have been prevalent during the last month of the quarter. One case of cholera occurred at Ilkeston, Nottinghamshire; continued fever has been very prevalent; diarrhœa has been very common; scarlatina prevailed extensively. At Runcorn, Cheshire, cholera and diarrhœa. Diarrhœa, it is said, has prevailed to an unusual extent during the whole of September; and all the deaths from cholera have taken place in that month. At Greengate, Lancashire, there have been eighty deaths from diarrhœa. In other parts of England the same prevalence of diarrhœa, fever, etc., is remarked, proving, beyond any doubt, the very intimate connexion between cholera and fever.

Morton notices the connexion of cholera with endemic fevers of various types; and Dr. Armstrong described cases similar to those witnessed during the present epidemic under the head of congestive typhus. Dr. Charles W. Bell, after becoming acquainted with cholera in this country, treated it in Persia, and he says that in that country the disease was ushered in by a regular succession of epidemics, commencing in a fever, apparently continued, but changing into a remittent or intermittent type; the cold stage of these types of fever frequently assuming all the appearances of cholera. This disappeared, and the epidemic resumed the character of remittent or continued for fever a time. Having thus witnessed, he adds, continued fever gradually resolving itself into cholera, and choleia into continued fever, by slow gradations, I am consequently inclined to consider cholera as an aggravated form of fever. Dr. Bird also observed this analogy and intimate connexion of the two diseases: he says that several cases came under his observation in India, where cholera and fever alternated with each other. It is, therefore, a fact well authenticated by both Indian and European practitioners, that remittent and continued fever not unfrequently precedes, accompanies, and follows cholera as an epidemic, even at long periods before and after the appearance of the major malady, consequently it is of the utmost importance, as Dr. Macloughlin justly observes, that everything connected with the public health, before, during, and after epidemic cholera, ought not to be overlooked, as such facts, well recorded, may lead us to the knowledge of what is the primary cause of epidemic cholera.

To conclude: it appears to me, that cholera is the result of a poison acting on the organism, and more particularly on the sympathetic system of nerves, in the same manner as that which produces remittent and continued fever, differing only in degree. If the poison producing both forms of the disease is not the same, there is at least a very great analogy in their effects. We frequently observe cases of continued fever which resemble the second stage of cholera, and which prove fatal before any reaction takes place; when, however, reaction does take place in cholera, we observe all the symptoms of continued fever. The excessive diarrhœa is but an effort of nature to eliminate the poison and its effects; we frequently observe a similar effort, for a similar purpose, and from a similar cause, but in a minor degree, in continued fever. It is reasonable to suppose that when the system is under the influence of a poison possessing a high degree of intensity, that the elimiating process will be of corresponding severity; and as the intestinal canal is the great emunctory or outlet which nature has provided for that purpose, we cannot be surprised at the amount of purging and vomiting in cases of cholera, nor ought those symptoms to weigh against the identity of the cause of this disease and remittent and continued fever. The intimate nature of the poison which produces continued fever is unfortunately beyond our powers of investigation, yet the great analogy, if not perfect identity, of cholera and fever, seems to point to a common origin; their *seeming* differences being owing to intensity of cause, with contingent and concurring circumstances.

---

